# Entropy in Landscape Ecology: A Quantitative Textual Multivariate Review

**DOI:** 10.3390/e23111425

**Published:** 2021-10-28

**Authors:** Samuel A. Cushman

**Affiliations:** USDA Forest Service, Rocky Mountain Research Station, Flagstaff, AZ 86001, USA; Samuel.cushman@usda.gov

**Keywords:** entropy, landscape, review, multivariate textual analysis, pattern

## Abstract

This paper presents a multivariate textual analysis of more than 1300 papers on entropy in ecology. There are six main insights that emerged. First, there is a large body of literature that has addressed some aspect of entropy in ecology, most of which has been published in the last 5–10 years. Second, the vast majority of these papers focus on species distribution, species richness, relative abundance or trophic structure and not landscape-scale patterns or processes, pe se. Third, there have been few papers addressing landscape-level questions related to entropy. Fourth, the quantitative analysis with hierarchical clustering identified a strongly nested structure among papers that addressed entropy in ecology. Fifth, there is clear differentiation of papers focused on landscape-level applications of entropy from other papers, with landscape focused papers clustered together at each level of the hierarchy in a relatively small and closely associated group. Sixth, this group of landscape-focused papers was substructured between papers that explicitly adopted entropy measures to quantify the spatial pattern of landscape mosaics, often using variations on Boltzmann entropy, versus those that utilize Shannon entropy measures from information theory, which are not generally explicit in their assessment of spatial configuration. This review provides a comprehensive, quantitative assessment of the scope, trends and relationships among a large body of literature related to entropy in ecology and for the first time puts landscape ecological research on entropy into that context.

## 1. Introduction

There has long been a deep interest in applying the most fundamental laws of physics to explain and understand ecosystems, populations, communities and landscapes. In particular, for many decades, there have been efforts to apply the second law of thermodynamics and the entropy concept to topics as diverse as ecosystem structure [1], landscape geomorphology [2], urban geography [3], and biological evolution [4].

Several of the seminal works in the field of landscape ecology explicitly discussed entropy and thermodynamics, and suggested that future work should develop methods to quantify entropy and integrate pattern–process measurement with fundamental physical theory [5,6]. These papers treated entropy conceptually and made generalizations regarding its potential importance to the field and speculated about how landscape pattern may be related to entropy, such as suggesting that aggregated patterns have low entropy and dispersed patterns have high entropy (note that [7] showed, conversely, that landscapes that are aggregated and dispersed both have low entropy and entropy is highest in spatially random landscapes). Other early landscape ecology papers also discussed the attractive idea of a conceptual foundation for landscape ecology in thermodynamics generally and entropy particularly [6,8,9]. However, since the late 1980s, there has been, until recent few years, a prolonged drought in entropy research in landscape ecology, with a few exceptions (see [10,11,12]).

An entropy renaissance in landscape ecology, of a sort, began with the review paper by Vranken [13], who presented an overview of the use of entropy in landscape ecology and identified three main uses of the entropy concept in past landscape ecology research, including spatial heterogeneity, unpredictability of pattern dynamics, and pattern dependence on scale. They concluded from their review that thermodynamic interpretations of spatial heterogeneity in the literature are not relevant, that thermodynamic interpretations related to scale dependence are highly questionable and that of all applications of entropy in landscape ecology, only unpredictability could be thermodynamically relevant if appropriate measurements were performed to test it.

The assertion that thermodynamic interpretations of heterogeneity were not relevant motivated Cushman [7,14,15] to demonstrate the utility of Boltzmann entropy for measuring landscape patterns. Cushman [15] demonstrated that the entropy of a landscape mosaic of patches could be measured as the logarithm of the number of arrangements that could be obtained (microstates) that produce the same microstate (edge length). Importantly, he showed that entropy is maximized in the most probable arrangement, corresponding to randomness, and that both spatially clumped and dispersed patterns have low entropy. Cushman [7] further demonstrated the generality of this method and its practical application to large landscapes with many patch types. This inspired a number of recent papers evaluating different measures of landscape configurational entropy [16,17,18,19,20,21,22].

Recently, [18] conducted a review of applications of Boltzmann entropy measures of landscape structure and configuration. They identified two main approaches to Boltzmann entropy in landscape ecology that were developed from distinct ideas, apply to different landscape models and result in different Boltzmann entropies. They proposed a general method for both patch mosaics and gradient models and theoretically analyzed and experimentally tested them. They found challenges in each possible approach for a generalized formulation of landscape entropy and proposed further work to develop an ideal method, which they pursued in [23] producing a Boltzmann entropy measure for landscape structure that appears to be fully thermodynamically consistent.

Despite recent interest in landscape entropy (e.g., [7,14,15,16,17,18,19,20,21,22,23]), including recent review papers (e.g., [13,18]) there has not yet been a comprehensive, quantitative review of a broad and representative sampling of the literature addressing entropy issues in ecology. Such a broad review using objective and quantitative methods would be very useful to understand the scope of work, its evolution and areas of focus and recent innovation. This, in turn, is important to guide future work into the areas of the most importance and potential benefit. The purpose of this paper is to present a multivariate textual analysis of a large sample of literature on entropy in ecology to put landscape ecological research on entropy into a broader context and to elaborate on the scope and direction of recent research trends.

## 2. Methods

### 2.1. Literature Search

I conducted a literature search in August 2021 in WoS (Web of Science). I used the features of the WoS search engine that provides groups of words and logical operators. Thus, the search was done according to the topic (which includes title, abstract, author keywords, and keywords plus) for the key phrases (“*entropy*” *AND* “*ecology*”). This was a purposefully broad search to cast a wide net for the full scope of work on entropy in ecology. The goal was to sample the full range of entropy research in ecology to provide a complete context to assess the status, trends and prospects for future development of landscape-level research on entropy. This search produced 1338 records. I downloaded the title, keywords, abstract and publication year of these records for use in subsequent statistical analyses.

### 2.2. Statistical Analyses

To assess the scope, trends and focal areas of entropy research in ecology generally and landscape ecology particularly, I conducted two complementary statistical analyses. The first one (cluster analysis) relied on intrinsic information associated with the papers (title, abstract, keywords) to quantify the interrelationships among papers in terms of the frequency of text words, which indicates relatedness of topic, method and focus. The second analysis (random forest) used as input outcomes of the cluster analysis and the frequency of text words among papers to quantify the meaning and relatedness of the clusters identified in the cluster analysis. This is a commonly recommended two-step approach that defines clusters based on their multivariate similarity and then post hoc quantifies their meaning with a method that discriminates between clusters based on their multivariate differences [24].

### 2.3. Clustering Analysis

I conducted a hierarchical agglomerative clustering analysis [24] to group the papers according to the frequency of individual words in their titles, abstracts and keywords. The analysis was intended to objectively identify the hierarchical grouping structure of papers based on the frequency of the words they use. The dataset for the clustering was prepared by turning each word into a dummy variable, and for each paper counting the number of times it occurred in the title, abstract and keywords. The matrix of word frequency across papers was then filtered to remove all words that occurred less than 30 times across all papers, and all words that were deemed to be not informative (e.g., particles, articles, adverbs, adjectives). Following the approach in [25] I computed the Bray–Curtis distance matrix on the word frequency matrix and then conducted hierarchical clustering [24] using Ward’s fusion method in R [26] using the hclust package. I then identified several levels of clustering for further evaluation based on the fusion pattern.

### 2.4. Random Forest Analysis

I then used random forest [27] to evaluate the relationship between the clusters identified and the textual terms contained in their member papers. Random forest is a machine learning algorithm that uses an ensemble of weak learners (bootstrapped classification or regression trees) to predict a continuous or categorical response variable. In this analysis I employed random forest to predict a categorical response variable (cluster membership) as a function of the frequency of unique words in the title, abstract and key words of the papers. Specifically, this analysis was done in two steps for each level of the clustering dendrogram. First, I ran the model improvement ratio (MIR; [28]) algorithm to select a parsimonious set of text terms as predictors of cluster membership and rank their predictive importance. The model improvement ratio provides a measure of the relative improvement in model prediction for each successive variable added to a random forest model, beginning with the most predictive variable and adding additional variables sequentially that maximize predictive ability in a forward-selection framework. I applied MIR to predict membership in each level of the cluster dendrogram (2, 3, 4 and 7 cluster solutions, see Results) based on the frequency of words within papers belonging to each cluster. This provides a means to interpret the meaning of the clusters in terms of what text terms distinguish them most strongly. Second, I predicted cluster membership with random forest using this suite of retained variables and used the standard classification table produced by the r package randomforest, which records omission and commission errors and the Out-of-Bag error for the classification, to measure the strength of discrimination among clusters in terms of their textual attributes.

### 2.5. Results

**Figure 1:**
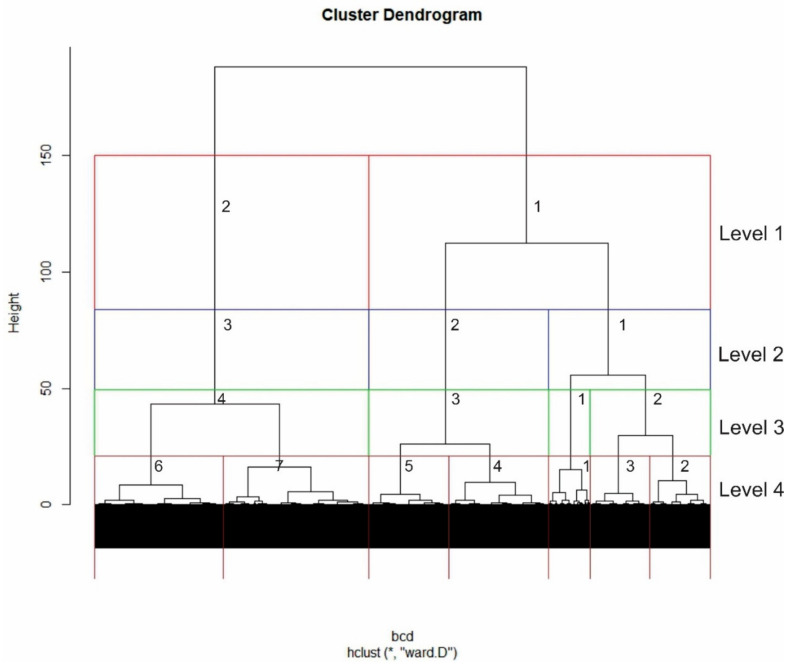
Hierarchical clustering dendrogram of 1338 “Entropy Ecology” papers based on Ward’s fusion on Bray–Curtis Distance among 997 title, keyword and abstract words. Colored boxes identify the hierarchical structure that is analyzed further: Red—Cluster 1 and Cluster 2; Blue—Cluster 1, 2 and 3; Green—Cluster 1, 2, 3 and 4; Cyan—Cluster 1, 2, 3, 4, 5, 6 and 7.

The hierarchical clustering clearly identified several nested levels of structure among “Ecology and Entropy” papers (Figure 1). There is a clear first level split at about Height = 125 which divides the papers into two highly distinct groups (Cluster 1 vs. Cluster 2 Level 1; Red Boxes Figure 1). A clear second level is also apparent in which Cluster 1 Level 1 is split into two sub-clusters (Cluster 1, Cluster 2 Level2), while Cluster 2 Level 1 remains undivided and becomes Cluster 3 Level 2 (Blue Boxes Figure 1). At level 3, Cluster 1 from Level 2 is split into two subclusters (becoming Cluster 1 and 2 Level 3), while Cluster 2 and 3 from Level 2 remain undivided and become Clusters 3 and 4 Level 3 (Green Boxes Figure 1). In the next, and last, level considered in this analysis, Clusters 2, 3 and 4 from Level 3 are split, producing a 7 cluster grouping (Cyan boxes Figure 1)

## 3. Cluster Interpretation

### 3.1. Cluster 1 and 2 Level 1

**Figure 2:**
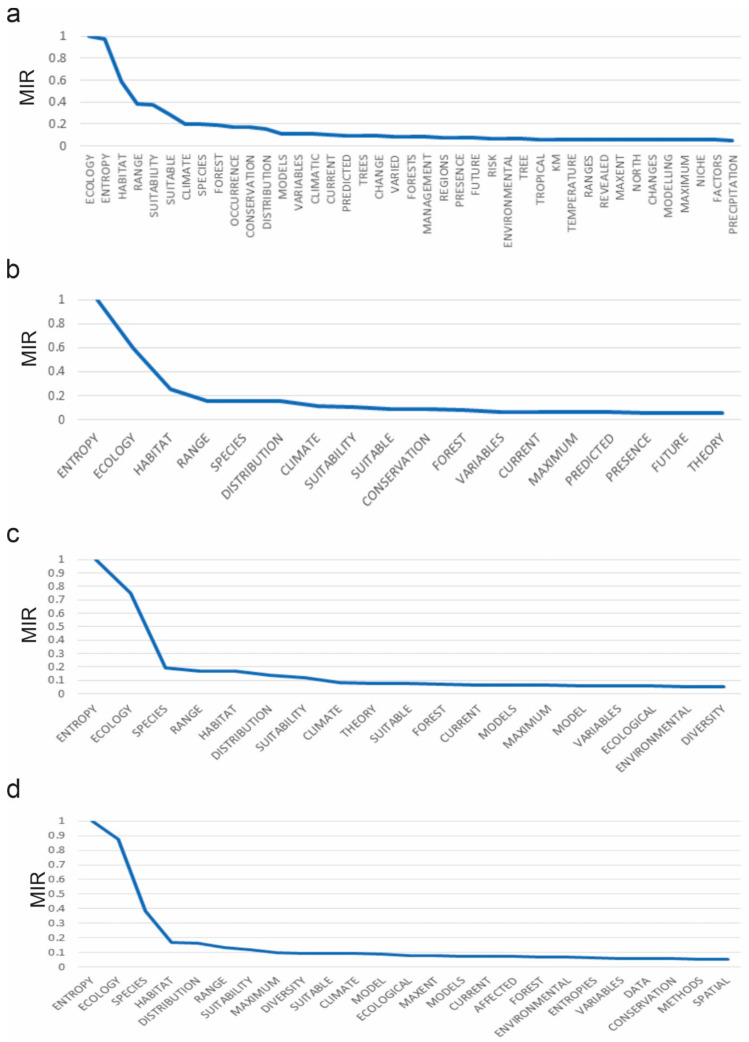
Ranked model improvement ratio (MIR) score for all title, abstract and keywords that had a greater than 0.05 MIR value for (**a**) the first level-random forest model, (**b**) the second-level random forest model, (**c**) the third-level random forest model, and (**d**) the fourth-level random forest model.

The random forest model improvement ratio variable selection procedure retained 246 words as predictor variables to predict Cluster 1 vs. Cluster 2 membership in the first level of the dendrogram (Table S1). A total of 41 words had a model improvement ratio effect of greater than 0.05 (>5% as large model improvement as the most predictive variable; Figure 2a).

The most important predictors were ECOLOGY and ENTROPY, which were both near 1 in MIR value, followed by HABITAT, RANGE, SUITABILITY, SUITABLE, CLIMATE, SPECIES, FOREST, OCCURRENCE, CONSERVATION, DISTRIBUTION, MODELS, VARIABLES, CLIMATIC, CURRENT, PREDICTED, which all had a greater than 0.1 MIR value (Figure 2a).

The random forest model using all 246 retained predictor words (Table S2) to predict membership of publications in Cluster 1 vs. Cluster 2 was quite successful, with an Out-of-Bag error of 23%. There was generally greater success in correctly assigning Cluster 1 publications (18.8% Class Error), than Cluster 2 (28.7% Class Error; Table S2).

**Figure 3:**
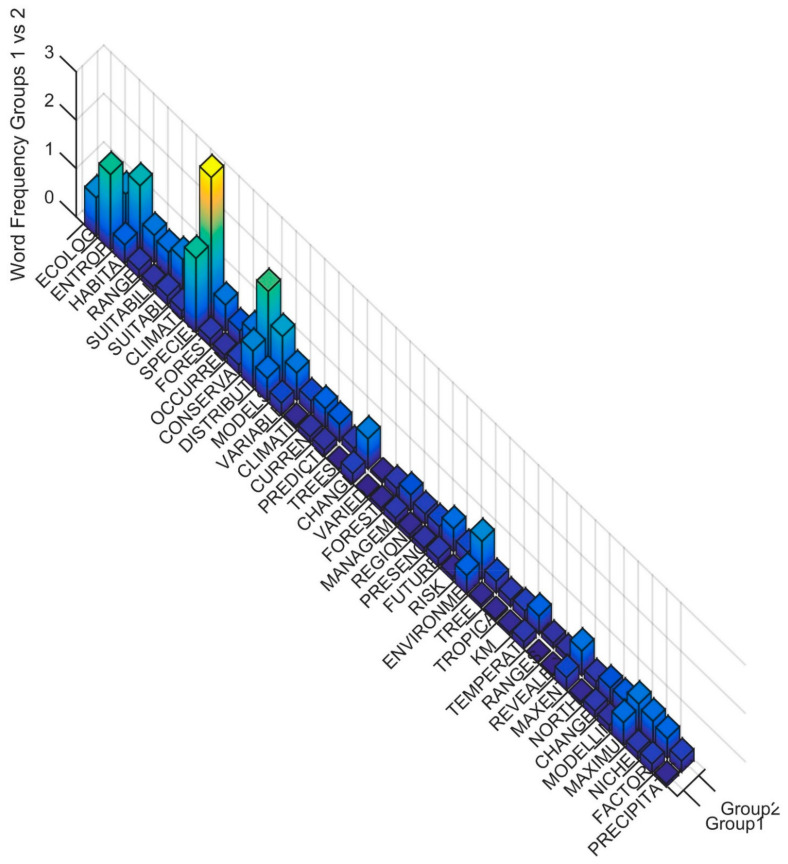
Word frequency for the most important predictor words distinguishing Cluster 1 vs. Cluster 2. The word order is from the most important predictor (ECOLOGY) to the least (PRECIPITATION), separated between Cluster 1 (Group1) and Cluster 2 (Group2). The Z axis records the word frequency of each term per paper in each cluster (e.g., a value of 1 means there is an average occurrence of the word 1 time in the title, keywords and abstract).

To interpret the differences in the Clusters in the terms used in the title, keywords and abstract, I produced rank order word frequency plots for the 46 words with MIR value greater than 0.05 (Figure 3). Based on this ranked word frequency plot I interpret these two main divisions as follows. Cluster 1 is distinguished by having relatively higher use of the terms ENTROPY and ECOLOGY than Cluster 2. This suggests that these papers are more formally focused on applications of entropy in ecology. In contrast, Cluster 2 has a large number of words with relatively high frequency of use. In particular, SPECIES, CONSERVATION, DISTRIBUTION, HABITAT, ENVIRONMENTAL, MAXENT, RANGE are relatively higher in frequency in Cluster 2 than Cluster 1. This suggests that Cluster 2 is focused on species distribution modeling using climatic and environmental variables as predictors and employing MAXENT methods, with frequent applications to conservation questions.

### 3.2. Cluster 1, 2 and 3 Second Level

In the second level of the hierarchical clustering, the previous Cluster 2 is unchanged, indicating that the papers focusing species distribution using MAXENT are deeply different in the terms used in title, keywords and abstract than the other Ecology and Entropy papers (Figure 1). In contrast, the previous Cluster 1 is split, suggesting a dichotomy in the papers focused explicitly on entropy in ecology. The random forest model improvement ratio variable selection procedure retained 246 words as predictor variables to predict Cluster 1, Cluster 2 and Cluster 3 membership in the second level of the hierarchical clustering (Table S3). A total of 18 words had a model improvement ratio effect of greater than 0.05 (>5% as large model improvement as the most predictive variable). These are show in decreasing rank order of model improvement ratio in Figure 2b.

The most important predictor of membership among Clusters 1, 2 and 3 in level 2 was ENTROPY, which had a MIR value nearly twice that of the next highest predicting term (ECOLOGY; Figure 2b). These were followed, in order of decreasing added predictive ability, by HABITAT, RANGE, SPECIES, DISTRIBUTION, CLIMATE, SUITABILITY, SUITABLE, CONSERVATION, FOREST, VARIABLES, CURRENT, MAXIMUM, PREDICTED, PRESENCE, FUTURE and THEORY, which all had a greater than 0.05 MIR value.

The random forest model using all 246 retained predictor words (Table S4) to predict membership of publications in the three clusters at level 2 was quite successful, with an Out-of-Bag error of 33%. There was generally greater success in correctly assigning Cluster 1 publications (18.7% Class Error) and Cluster 3 (16.6% Class Error), than Cluster 2 (72.1% Class Error; Table S4). Cluster 2 was often misassigned to Cluster 3 by random forest.

**Figure 4:**
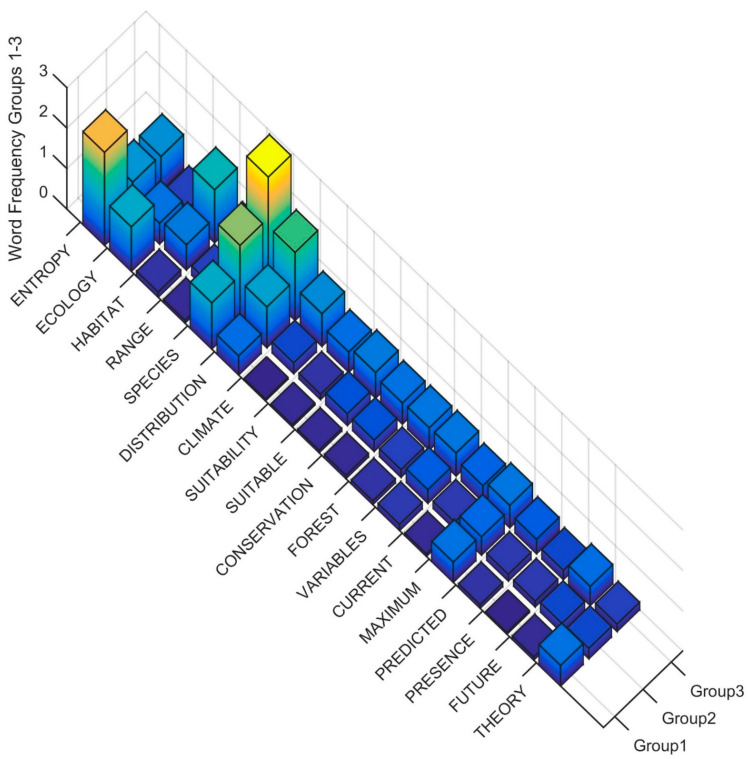
Word frequency for the most important predictor words distinguishing clusters in the second level of the dendrogram. The word order is from the most important predictor (ENTROPY) to the least (THEORY), separated between Clusters (Group1, Group2 ad n Group3). The Z axis records the word frequency of each term per paper in each cluster (e.g., a value of 1 means there is an average occurrence of the word 1 time in the title, keywords and abstract).

To interpret the differences in the Clusters at the second level of the dendrogram in the terms used in the title, keywords and abstract, I produced rank order word frequency plots for the 18 words with MIR value greater than 0.05 (Figure 4). Based on this ranked word frequency plot, Cluster 1 is distinguished by having relatively higher use of the terms ENTROPY, ECOLOGY and THEORY than Clusters 2 and 3. This suggests that the papers in Cluster 1 are more formally focused on theoretical applications of entropy in ecology. In contrast, Cluster 2 and Cluster 3 have relatively higher use of SPECIES, DISTRIBUTION, CLIMATE, HABITAT, FUTURE, and CLIMATE, with Cluster 3 having higher usage of all of these terms as well as CURRENT, PREDICTED, SUITABILITY. This suggests that Cluster 2 and 3 in the second level of the dendrogram both include papers that focus on species distribution modeling, but that Cluster 3 is more coherently focused on species habitat and distribution modeling with MAXENT and projecting future changes, particularly as a result of climate change.

### 3.3. Cluster 1, 2, 3 and 4 Third Level

In the third level of the hierarchical clustering, the previous Clusters 2 and 3 are unchanged, again indicating that the species distribution papers using MAXENT are deeply different in the terms used in title, keywords and abstract than the other Ecology and Entropy papers (Figure 1). The random forest model improvement ratio variable selection procedure retained 246 words as predictor variables to predict Cluster 1, Cluster 2, Cluster 3 and Cluster 4 membership in the third level of the hierarchical clustering (Table S5). A total of 18 words had a model improvement ratio effect of greater than 0.05 (5% as large model improvement as the most predictive variable). These are show in decreasing rank order of model improvement ratio in Figure 2c.

The most important predictor of membership in the third level of the dendrogram was ENTROPY, which had a MIR value approximately 67% higher than the next highest predicting term (ECOLOGY; Figure 2c). These were followed, in order of decreasing added predictive ability, by SPECIES, RANGE, HABITAT, DISTRIBUTION, SUITABILITY, CLIMATE, THEORY, SUITABLE, FOREST, CURRENT, MODELS, MAXIMUM, MODEL, VARIABLES, ECOLOGICAL, ENVIRONMENTAL, and DIVERSITY, which all had a greater than 0.05 MIR value.

The random forest model using all 246 retained predictor words (Table S6) to predict membership of publications in the four clusters in level 3 was quite successful, with an Out-of-Bag error of 38.3%. There was high success in correctly assigning Cluster 4 publications (14.6% Class Error) as compared to Clusters 1, 2, 3 (48.9%, 43.3%, and 68.5% Class Error, respectively; Table S6). Cluster 1 was often misassigned by random forest to Cluster 2, while Cluster 2 was often misassigned to Clusters 3 and Cluster 4. Cluster 3 was most frequently misassigned to Cluster 4, and Cluster 4 most frequently to Cluster 3.

**Figure 5:**
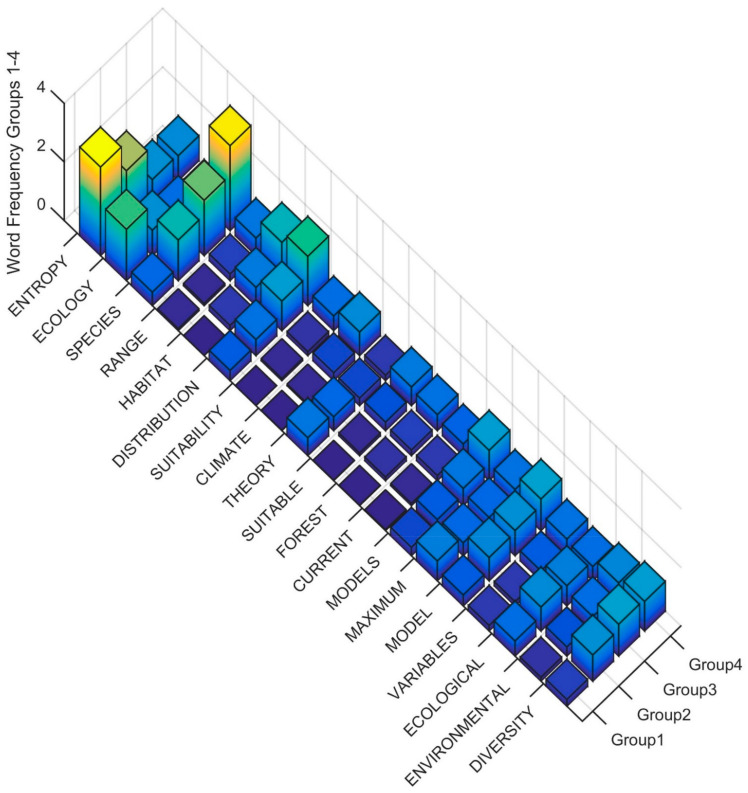
Word frequency for the most important predictor words distinguishing clusters in the third level of the dendrogram. The word order is from the most important predictor (ENTROPY) to the least (DIVERSITY separated between Clusters (Group1, Group2, Group3, Group4). The Z axis records the word frequency of each term per paper in each cluster (e.g., a value of 1 means there is an average occurrence of the word 1 time in the title, keywords and abstract).

To interpret the differences in the Clusters in level three of the dendrogram in the terms used in the title, keywords and abstract, I produced rank order word frequency plots for the 18 words with MIR value greater than 0.05 (Figure 5). Based on this ranked word frequency plot, Cluster 1 is distinguished by having relatively higher use of the terms ENTROPY, ECOLOGY and THEORY than Clusters 2, 3 and 4. This suggests that the papers in Cluster 1 are more formally focused on theoretical applications of entropy in ecology. In contrast, Clusters 2, 3 and 4 have relatively higher use of SPECIES, DISTRIBUTION, CLIMATE, HABITAT, FUTURE, CLIMATE, with Cluster 4 having higher usage of all of these terms as well as CURRENT, PREDICTED, SUITABILITY. The term DIVERSITY is also in high frequency in Clusters 2, 3 and 4 as compared to Cluster 1, suggesting that these clusters also contain many papers focused on community structure and species diversity using entropy-based methods. This suggests that Clusters 2 and 3 in the third level of the dendrogram both include papers that focus on species distribution and community structure using entropy-based methods, but that Cluster 4 is more coherently focused on species habitat and distribution modeling with MAXENT and projecting future changes, particularly as a result of climate change.

### 3.4. Cluster 1, 2, 3, 4, 5, 6, and 7 Fourth Level

In the fourth level of the hierarchical clustering, the previous Clusters 2, 3 and 4 are split, and Cluster 1 from the third level is unchanged (Figure 1 Cyan boxes). The random forest model improvement ratio variable selection procedure retained 246 words as predictor variables to predict membership in the seven clusters at the fourth level of the hierarchical clustering (Table S7). A total of 25 words had a model improvement ratio effect of greater than 0.05 (>5% as large model improvement as the most predictive variable). These are show in decreasing rank order of model improvement ratio in Figure 2d.

The most important predictor of membership in the fourth level of the dendrogram was ENTROPY, which had a MIR value approximately 15% higher than the next highest predicting term (ECOLOGY; Figure 2d). These were followed, in order of decreasing added predictive ability, by SPECIES, HABITAT, DISTRIBUTION, RANGE, SUITABILITY, MAXIMUM, DIVERSITY, SUITABLE, CLIMATE, MODEL, ECOLOGICAL, MAXENT, MODELS, CURRENT, FOREST, ENVIRONMENTAL, ENTROPIES, VARIABLES, DATA, CONSERVATION, METHODS, and SPATIAL, which all had a greater than 0.05 MIR value.

The random forest model using all 246 retained predictor words (Table S8) to predict membership of publications in the fourth level of the dendrogram was only marginally successful, with an Out-of-Bag error of 60.4%. There was high success in correctly assigning Cluster 1 (22.8% Class Error) and Cluster 7 (29.1% Class Error) as compared to Clusters 2, 3, 4, 5, 6 and 7 (62.1%, 89.9%, 69.4%, 92.5% and 66.7% Class Error, respectively). Cluster 1 was most often misassigned by random forest to Cluster 2, while Cluster 7 was most often misassigned to Cluster 6 or 4 and Cluster 3 to Cluster 4.

**Figure 6:**
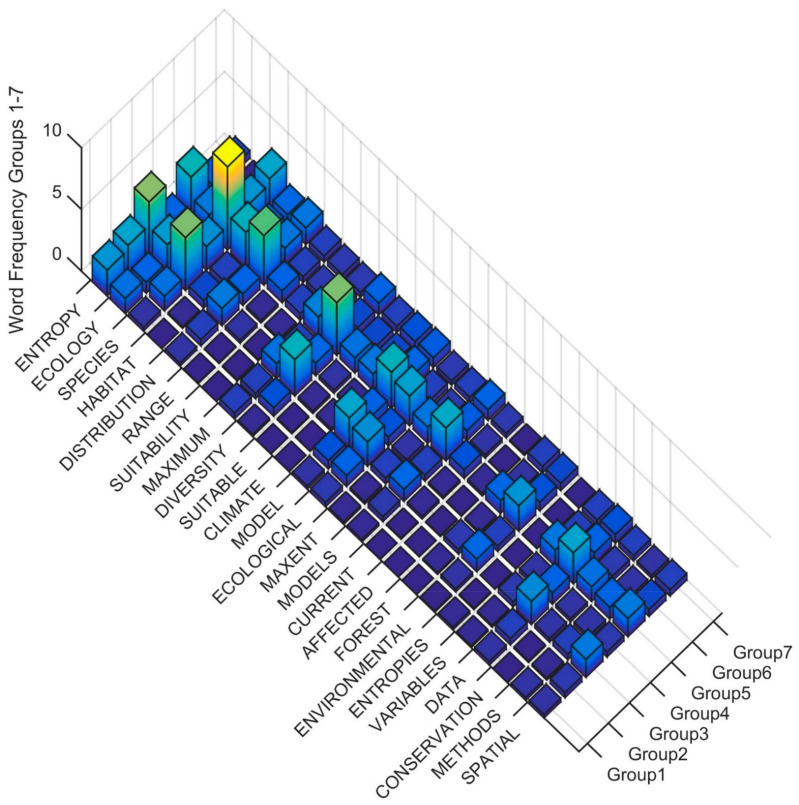
Word frequency for the most important predictor words distinguishing clusters in the fourth level of the dendrogram. The word order is from the most important predictor (ENTROPY) to the least (SPATIAL) separated between Clusters (Group1, Group2, Group3, Group4, Group5, Group6, Group7). The Z axis records the word frequency of each term per paper in each cluster (e.g., a value of 1 means there is an average occurrence of the word 1 time in the title, keywords and abstract).

To interpret the differences in the Clusters in level four of the dendrogram in the terms used in the title, keywords and abstract, I produced rank order word frequency plots for the 25 words with MIR value greater than 0.05 (Figure 6). Based on this ranked word frequency plot Clusters 1, 2 and 3 are distinguished by having relatively higher use of the term ENTROPY, and Cluster 1 is distinguished by not also having high frequency of SPECIES, HABITAT, DIVERSITY, DISTRIBUTION, RANGE. This suggests that the papers in Cluster 1 are more formally focused on applications of entropy in studies that are not addressing species distribution or community structure with entropy-based methods. In contrast, Clusters 2 through 7 have relatively higher use of SPECIES, DISTRIBUITON, CLIMATE, HABITAT, FUTURE, CLIMATE, with Cluster 4 having higher usage of all of these terms as well as CURRENT, PREDICTED, SUITABILITY. The term DIVERSITY is also in high frequency in Clusters 3 and 5 as compared to other clusters, suggesting that these clusters contain many papers focused on community structure and species diversity using entropy-based methods.

## 4. Temporal Trends

### 4.1. Cluster 1 vs. Cluster 2

**Figure 7:**
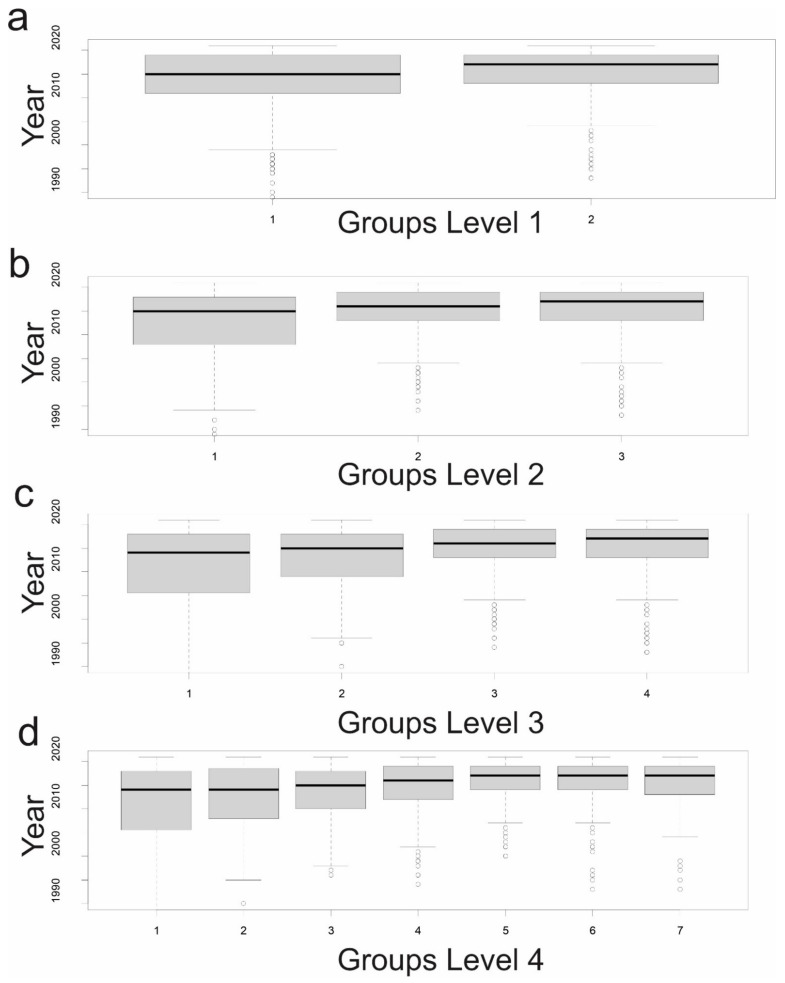
Boxplot of publication dates between papers in (**a**) level 1, (**b**) level 2, (**c**) level 3 and (**d**) level 4 of the dendrogram.

At the first level of the clustering, Cluster 1 and Cluster 2 are temporally overlapping to a relatively high degree (Figure 7a), with Cluster 1 being a temporally longer group which started earlier (first papers in the 1960s) and has a lower median publication year (2015), while Cluster 2 is more recent (first paper in 1994) and a higher median publication year (2018).

### 4.2. Cluster 1, 2 and 3 Level 2

In the second level of the dendrogram, Cluster 2 from level 1 is split into two clusters (Clusters 1 and 2 in level 2). The first of these (Cluster 1) contains papers that are relatively older and published over a wider period of time (median 2015 and interquartile range 2018–2009; Figure 7b). Cluster 2 contains more recent papers (median 2017) with the first paper published in 1995. Cluster 3 in the second level is unchanged from Cluster 2 in the first.

### 4.3. Clusters 1, 2, 3 and 4 Level 3

In the third level of the dendrogram, Cluster 1 from the second level is split and the previous Clusters 2 and 3 remain unchanged (now Clusters 3 and 4). The division of this cluster again partly separates older from newer publications (Cluster 1 median publication year 2013 vs. Cluster 2 2014) with Cluster 1 having all the oldest papers (including all before 1990, Figure 7c).

### 4.4. Clusters 1, 2, 3, 4, 5, 6, 7 Level 4

In the fourth level the previous level, Clusters 1, 3 and 4 are split. There is clear temporal structure with Cluster 1 being oldest and the widest temporal breadth, followed by Cluster 2, 3 and 4 (Figure 7d). Cluster 7 is next most recent (based on median paper publication date). Clusters 5 and 6 are most recent, with median publication date after 2010. Cluster 5 is most recent, with no papers before 2003.

## 5. Landscape Ecology and Entropy among Clusters

The previous results focus on the full structure of all papers found in the Web of Science search using “Entropy” and “Ecology” as search terms. This review focuses on the emergence and evolution of entropy concepts and methods in landscape ecology. The purpose of the prior results is to show the full context of entropy research in ecology which is necessary to put landscape ecology entropy research into context. This section presents the frequency of key “Landscape Ecology” words across the cluster structure described above to identify the areas of landscape ecology research related to entropy within the full context of ecological entropy work. The key words I focus on for identifying landscape ecology applications of entropy include: SPATIAL, LANDSCAPE, STRUCTURE, SCALE, PATTERN, SHANNON, and BOLTZMANN. I chose LANDSCAPE as a key word because it identifies papers that explicitly have a landscape focus. SPATIAL, STRUCTURE and PATTERN were chosen because they identify papers that focus on the structure and configuration of landscapes, rather than the structure of nonspatial data (as is the case in most community ecology applications that focus on species abundance, species richness, and community structure). I include SHANNON and BOLTZMANN as they are the two main “kinds” of entropy that are used in landscape ecology research, with Shannon entropy as developed in information theory being a commonly used, but not spatially explicit [17] method, and Boltzmann entropy being more recently employed to explicitly focus on spatial patterns and their entropy in a microstate counting framework (e.g., [7,15,18]).

### 5.1. Clusters 1 vs. 2 Level 1

**Figure 8:**
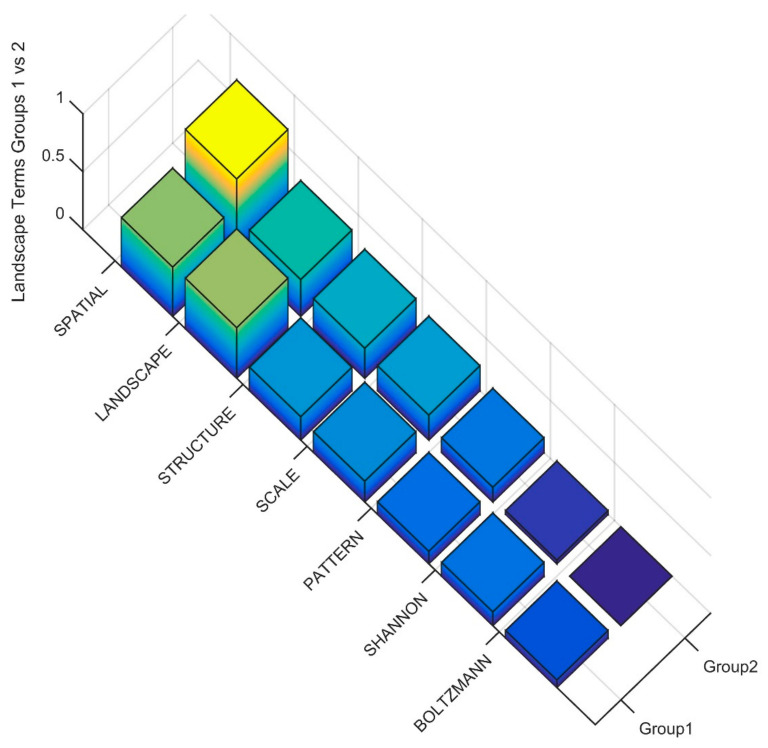
Frequency of key landscape ecology words among publications in Clusters 1 and 2 in level 1 of the dendrogram.

In the first-level major division between Cluster 1 and Cluster 2, there are clear differences in the frequency of key landscape ecology terms (Figure 8). Most importantly, we see that the term LANDSCAPE is more frequent in Cluster 1, indicating that Cluster 1 contains more papers with an explicit landscape ecology focus. Additionally, SHANNON and BOLTZMANN are nearly exclusively confined to Cluster 1, indicating that Cluster 1 is where the papers focused on calculating the entropy of landscapes as a spatial attribute are concentrated.

### 5.2. Clusters 1–3 Level 2

**Figure 9:**
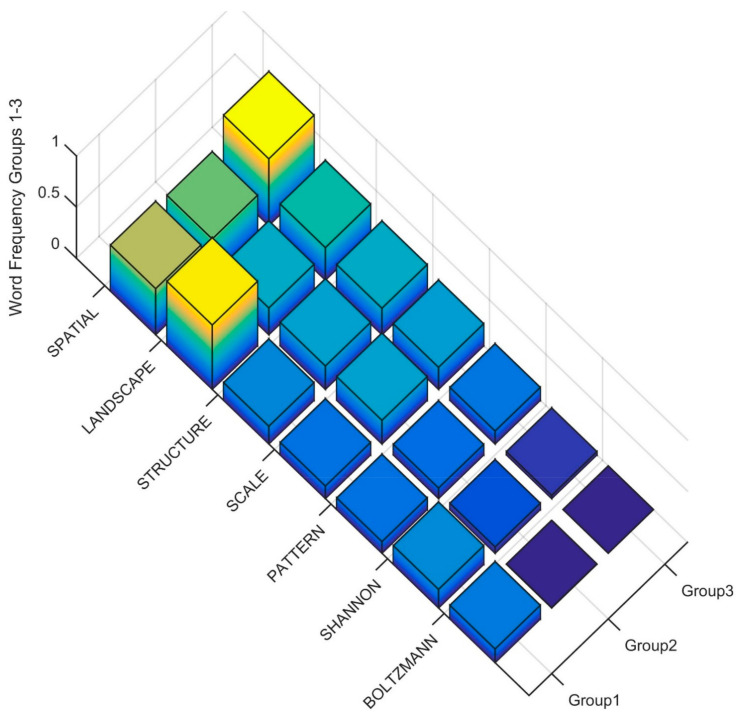
Frequency of key landscape ecology words among publications in Clusters 1–3 in level 2 of the dendrogram.

At the second level of the dendrogram we see further distinction among clusters in terms of the frequency and type of use of key “Landscape Ecology” terms (Figure 9). In particular, Cluster 1 has a high concentration of the term LANDSCAPE and nearly exclusive use of the term SHANNON, and fully exclusive use of the term BOLTZMANN. This indicates that landscape ecology research, particularly that focusing on calculating compositional (SHANNON) and configurational (BOLTZMANN) entropy is concentrated in Cluster 1 of the second tier.

### 5.3. Clusters 1–4 Level 3

**Figure 10:**
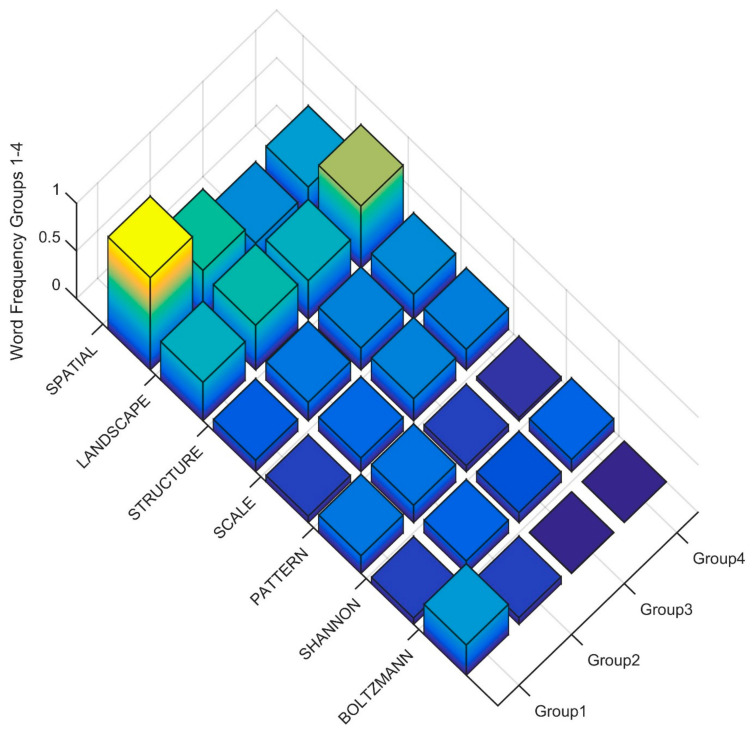
Frequency of key landscape ecology words among publications in Clusters 1–4 in level three of the dendrogram.

At the third level of the dendrogram there is clear distinction among the two different families of landscape ecology entropy research, with those focused on Boltzmann configurational entropy concentrated in Cluster 1 and those focused on Shannon entropy concentrated in Cluster 2 (Figure 10). Cluster 4 also includes frequent use of the term Shannon, but it is in applications to information entropy to measure aspects of community and ecosystems structure in a non-spatial, non-landscape context. Cluster 1 could be described based on the word frequency as “Spatial analysis of landscape structure and pattern using Boltzmann entropy,” while Cluster 2 could be described as including “Landscape spatial structural analysis using Shannon entropy.” The key distinction here is the term SPATIAL which is explicit in the Boltzmann entropy research but is not separated from composition in the Shannon entropy work.

### 5.4. Clusters 1–7 Level 4

**Figure 11:**
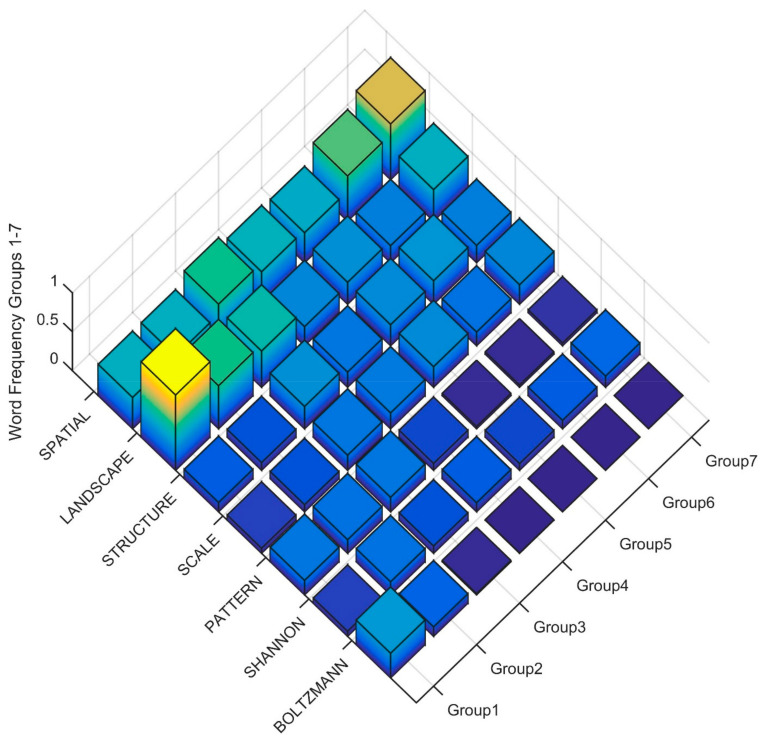
Frequency of key landscape ecology words among publications in Clusters 1–7 in level 4 of the dendrogram.

At the fourth level, there is clear concentration of key landscape ecology terms focused on entropy, with Cluster 1 having the largest concentration of the term LANDSCAPE and BOLTZMANN, with Cluster 2 also high in those terms (Figure 11). The term SHANNON is distributed at moderate frequency from Clusters 2 to 7, with highest frequency in Cluster 7 and also Cluster 2, indicating a relatively broader use of SHANNON in papers across the full scope of ecological entropy research, likely because information entropy measured by the Shannon index is common in community ecology studies as well as landscape ecology work focused explicitly on spatial patterns at the landscape level.

## 6. Discussion

Review papers have long been important in synthesizing past work and guiding future progress in scientific research. Most past literature reviews have been qualitative, based largely on the authors’ subjective thoughts after they have compiled and read a large number of papers on a topic (e.g., [29]). While this provides a means for authors to summarize their impressions about the state and trends in a field, it is limited by potential bias, representativeness and lack of quantitative measures. Other authors have advocated using quantitative methods, such as hierarchical ordering of papers across a priori categories (e.g., [30]), or, more recently, quantitative multivariate analysis, such as with hierarchical clustering on the attributes of a paper (e.g., [25]). This paper is the first review of ecological thermodynamics which utilizes a novel approach to conduct a rigorous, quantitative analysis of a large body of literature using textual analytics and multivariate statistics.

By compiling the titles, keywords and abstracts of all papers that were identified in a Web of Science search using the terms “Ecology” and “Entropy” I obtained a large database of literature on the subject of entropy in ecology. This produced a database of 1338 publications, which is more than can be reasonably or consistently read and summarized by traditional qualitative literature review methods. However, by treating unique words as variables and counting the number of occurrences of each unique word in the title, keywords and abstracts of each publication it was possible to produce a large quantitative database for textual analysis. Employing hierarchical clustering on a distance matrix produced from this table of word frequency enabled me to parsimoniously and unbiasedly describe the major patterns and relationships among papers that have addressed entropy in ecology.

This paper focuses on the uses and applications of entropy in landscape ecology. I purposely included a broad search (entropy and ecology) to put the landscape entropy work into its full context within the body of entropy work in ecology. There are six main insights that emerged from this analysis which are novel and have not been previously described by the other reviews that have recently been completed on entropy in landscape ecology (e.g., [13,18]), which are elaborated upon in the paragraphs below.

The first important insight is that there is a large body of literature that has addressed some aspect of entropy in ecology (more than 1300 papers), most of which have been published in the last 5–10 years. Indeed, there has been an enormous emerging interest in entropy in ecology, as indicated by the number of publications. Each of the main clusters of papers identified in this analysis has a median publication date after 2015, and several are comprised of papers from the last three to four years. This shows that there is an ongoing and broad-based expansion of research on entropy in ecology, spanning from species distribution, to macro and community ecology, to landscape ecology.

The second insight is that the vast majority of papers found in the literature search in this review focus either on species distribution modeling using MAXENT methods or use MAXENT or Shannon/information entropy to describe patterns of ecosystem and community structure, such as productivity, species richness, relative abundance or trophic structure (the Maximum Entropy Theory of Ecology (METE)). These papers use maximum entropy algorithms to predict species occurrence patterns or community structure, and generally are not explicitly assessing landscape-level, pattern–process relationships or using entropy to quantify of measure spatial patterns, per se. These two broad groups together comprise approximately 90% of the identified literature, with the species distribution modeling with MAXENT comprising approximately 60% and the METE group 30%.

Third, there have been few papers, relatively speaking, addressing landscape-level questions related to entropy. For example, only 148 of 1338 (approximately 10%) of publications found in this search include the term “landscape” in the title, abstract or keywords. The cluster analysis identified a group of approximately 90 papers that were closely focused on spatial entropy at the landscape level, comprising only approximately 7% of all the literature on entropy in ecology. This suggests that landscape ecological research on entropy is very limited. Indeed, the key papers developing and applying entropy concepts and methods in landscape ecology are very recent, published since 2015, and represent a new paradigm of research that has just begun to innovate.

The cluster analysis, however, clearly shows that this newly rediscovered focus on spatial structure and configuration in a thermodynamic context is closely related to much older research from the 1960s. Indeed, the broad cluster to which the landscape ecology entropy literature is grouped into has the largest temporal spread and earliest median publication date of all clusters of entropy in ecology papers identified in this review. For example, Leopold and Langbein [2] presented the “concept of entropy in landscape evolution”, and proposed that landscapes evolve toward maximization of entropy, measured by the most probable condition of the landscape with regard to the distribution of matter and energy, and proposed a series of equations to measure and predict this in hydrological systems. This focus on landscape evolution and maximization of entropy has been rekindled in recent research on calculating Boltzmann configurational entropy of landscapes (e.g., [14,15,17,22]), but this new focus is best seen as the continuation of an older line of work on entropy in ecology, which formally focused on thermodynamic and structural aspects of ecosystems and landscapes.

The fourth important insight from this review is that the quantitative analysis with hierarchical clustering identified a strongly nested structure among papers that addressed entropy in ecology. The first major division based on the deepest differences among papers divided those focused on theoretical applications of entropy in ecology from those focusing on species distributions, predictions of range shift and climate effects using MAXENT modeling. The second level of hierarchical structure further differentiated papers focusing on theoretical applications of entropy in ecology from two other larger groups of papers that focus on species distribution modeling and the Maximum Entropy Theory of Ecology, with one cluster focused more coherently on species habitat and distribution modeling with MAXENT and projecting future changes, particularly as a result of climate change, and the other more fully on community ecology and macroecology subjects. Similarly, the third level of clustering identified four distinct clusters, with Cluster 1 having much higher frequency of the terms ENTROPY, ECOLOGY and THEORY than Clusters 2, 3 and 4. This suggests that the papers in Cluster 1 are more formally focused on theoretical applications of entropy in ecology. In contrast, Clusters 2, 3 and 4 have relatively higher use of SPECIES, DISTRIBUTION, CLIMATE, HABITAT, FUTURE. The term DIVERSITY is also in high frequency in Clusters 2, 3 and 4 as compared to Cluster 1, suggesting that these clusters also contain many papers focused on community structure and species diversity using entropy-based methods.

Fifth, a post hoc analysis of the frequency of a selection of focused “landscape ecology” terms clarified where in this full structure of entropy research in ecology the landscape ecology focused papers fall. This showed a strong and clear differentiation from other papers, with landscape-focused papers clustered together at each level of the hierarchy in a relatively small and closely associated group.

Sixth, this group of landscape-focused papers was substructured between papers that explicitly adopted entropy measures to quantify the spatial pattern of landscape mosaics, often using variations on Boltzmann entropy, versus those that utilize Shannon entropy measures from information theory, which are not generally explicit in their assessment of spatial configuration. The application of Boltzmann entropy to landscape ecology questions in general and measuring landscape configuration in particular was first developed by [15] and extended and generalized by [7]. Subsequently, there has been rapid development of several alternative versions of Boltzmann entropy for measuring landscape patterns (e.g., [7,15,16,17,18,19]), with recent interest in applications of Wasserstein entropy [20,21,22] in measuring landscape patterns.

## 7. Future Directions and Applications

This review provides a novel view of the entropy research “landscape”. By conducting a large and representative literature search and employing objective and quantitative textual analytics, I was able to summarize, analyze and synthesize a much larger body of literature than has formerly been reviewed in the topics of entropy in ecology. This allowed me to identify the main focal areas of work, their relationships, and their historical development. The broad context provided by such a review is important to guide researchers to understand the field, the different concepts, terminology and paradigms used by researchers in different “clusters”, and to understand where entropy research in landscape ecology is heading now.

Much of current landscape ecology entropy research is focused on the best ways to calculate the configurational entropy of landscapes (e.g., [7,15,16]) and how to generalize that across different conceptual models of landscape structure, such as across mosaics, gradients and point patterns [18,23,31]. This is indeed a critical area of focus, given that a rigorous and consistent set of metrics for measuring and analyzing the entropy of landscapes is foundational to theoretical development and integration of entropy with modeling of pattern–process relationships. In this volume [31] demonstrates that the direct application of the Boltzmann relation [7] to calculate landscape entropy is equally applicable to landscape patch mosaics, point patterns and gradients, and [32] shows that it is fully thermodynamically consistent. This would seem to suggest that the Cushman method [7,15,31,32] to directly apply the classic and iconic Boltzmann relation to measurements of landscape entropy might be advantageous given its simplicity, elegance and direct linkage to original formulations of Boltzmann entropy.

Reaching consensus on the best ways to calculate configurational entropy, however, is only the first step. It is a foundation for real scientific advances which must follow, in areas such as understanding pattern–process relationships from a thermodynamic point of view [14,15], developing physics-based models that predict landscape structure and evolution [2] and the emergence of structure and order at a landscape scale from the action of dissipative structures in open thermodynamic systems [14,33,34].

Entropy is the most fundamental attribute of physical and biological systems given the primacy of the second law of thermodynamics in governing the direction of all change in physical and biological systems. There has been much interest in entropy in ecology, and this review quantified the main branches of research and how they have emerged in the past decades. I believe the most interesting and important branch has been the least developed. Namely, there have been a plethora of papers using information-entropy based methods to describe ecological systems and predict species distributions, but relatively few papers have attempted to integrate the second law into prediction of pattern–process relationships at landscape scales. In addition, linkage between formal analysis of entropy of living and physical systems with additional topics in complexity theory, such as logical depth [35,36], which is a measure of complexity of a system based on the length or complexity of computer code required to simulate its numerical attributes and the processing time required to run this code. Intuitively, there should be a relationship between entropy and complexity, such as measured by logical depth. A system with maximum entropy would have the lowest level of complexity based on logical depth, given maximum entropy equates to maximum randomness, and computer code to simulate randomness is simple and quick to run. Maximum entropy, conversely, corresponding to high aggregation or systematic dispersion, would require more complex simulation processes, and therefore represent a system of greater logical depth.

Some members of the landscape ecology community question the importance or utility of entropy research, seeing it only as a basis for measuring patterns, of which there are already many in the field. However, seeing entropy research as the way to integrate landscape ecology with physics, particularly with the fundamental processes of thermodynamics, and how entropy, exergy and enthalpy are the currencies of dynamic dissipative structures, such as organisms, ecosystems and landscapes, makes it clear that entropy will be a paradigmatic focus of the maturation of landscape ecology into a predictive, theory-based science of pattern–process relationships.

## Data Availability

Not applicable.

## Supplementary Materials

The following are available online at https://www.mdpi.com/article/10.3390/e23111425/s1. Table S1. Model improvement ratio retained variables for predicting Cluster 1 vs. Cluster 2 in Level 1, Table S2. Cluster 1 vs. Cluster 2 Random Forest classification table. Horizontal is predicted and vertical is actual, Table S3. Model improvement ratio retained variables for predicting Cluster 1, Cluster 2 and Cluster 3 in Level 2, Table S4. Cluster 1, Cluster 2 and Cluster 3 Random Forest classification table. Horizontal is predicted and vertical is actual, Table S5. Model improvement ratio retained variables for predicting Cluster 1, Cluster 2, Cluster 3 and Cluster 4 in Level 3, Table S6. Level 3 Random Forest classification table. Horizontal is predicted and vertical is actual, Table S7. Model improvement ratio retained variables for predicting Cluster 1, Cluster 2, Cluster 3, Cluster 4, Cluster 5, Cluster 6 and Cluster 7 in Level 4, Table S8. Level four clustering Random Forest classification table. Horizontal is predicted and vertical is actual.
