# Supplementary material for: Entropy in Landscape Ecology: A Quantitative Textual Multivariate Review
Source: Entropy (Basel). 2021 Oct 28;23(11):1425. doi: 10.3390/e23111425 (PMC8625677; doi:10.3390/e23111425)
Supplement: Supplementary file 1 [file entropy-23-01425-s001.zip › entropy-1374137-supplementary.pdf]

Supplemental Material

Table S1. Model improvement ratio retained variables for predicting Cluster 1 vs Cluster 2 in Level 1.

	importance
ECOLOGY	1
ENTROPY	0.975083761
HABITAT	0.583855504
RANGE	0.381327978
SUITABILITY	0.371852916
SUITABLE	0.286210707
CLIMATE	0.202222938
SPECIES	0.19542443
FOREST	0.190246698
OCCURRENCE	0.173675071
CONSERVATION	0.170506159
DISTRIBUTION	0.1512788
MODELS	0.112765069
VARIABLES	0.111785995
CLIMATIC	0.105926533
CURRENT	0.105040169
PREDICTED	0.095083517
TREES	0.094506454
CHANGE	0.093496132
VARIED	0.082867763
FORESTS	0.082548036
MANAGEMENT	0.079317064
REGIONS	0.077392346
PRESENCE	0.07516502
FUTURE	0.072091936
RISK	0.069047214
ENVIRONMENTAL	0.068587422
TREE	0.063527147
TROPICAL	0.061200494
KM	0.059691575
TEMPERATURE	0.058051878
RANGES	0.057977023
REVEALED	0.056685274
MAXENT	0.056620725
NORTH	0.056007473
CHANGES	0.054949754
MODELLING	0.054136312
MAXIMUM	0.053662318
NICHE	0.053079988
FACTORS	0.053015115
PRECIPITATION	0.052069316
PREDICT	0.04912091

RESPONSES	0.048497311
CONDUCTED	0.047030494
LOCAL	0.046261962
FOUR	0.042167839
DEGREES	0.041650125
THEORY	0.041545754
INVASIVE	0.040941684
RECORDS	0.040481937
SPATIAL	0.040133831
H	0.039569051
AFFECTED	0.038971311
DENSITY	0.038302209
DECREASED	0.038289352
VEGETATION	0.037582353
MONITORING	0.037423558
ELEVATION	0.036729974
NATIONAL	0.036612633
PLANT	0.036383266
ADSORPTION	0.034718329
COASTAL	0.034439394
SCENARIOS	0.034050353
SOIL	0.033768771
LAND	0.032443481
SYSTEMS	0.032031118
COVER	0.031923303
EFFECTS	0.029577911
COMPOSITION	0.02865034
ASSESS	0.028442614
PREDICTOR	0.02843441
PRINCIPAL	0.028005325
CONTENT	0.027474563
COLLECTED	0.027352231
RESPOND	0.026269207
SITES	0.02626251
MOUNTAIN	0.026119376
SIGNIFICANTLY	0.025531238
SELECTED	0.025398283
COAST	0.02510439
BIOCLIMATIC	0.024738446
MAINLY	0.024588899
SHIFT	0.023992854
PROCESS	0.022901178
AFRICA	0.02288174
COMMUNITY	0.022326576
CORRELATION	0.02203796
RICHNESS	0.021785895

VARIATION	0.021767312
HABITATS	0.021597937
TEMPERATURES	0.021576967
LEAF	0.02135943
AVERAGE	0.021251065
PROJECTED	0.020794811
PREDICTIVE	0.020789314
LOCATION	0.020739244
ACOUSTIC	0.020615866
INDICES	0.020290718
DRY	0.020183634
AMERICA	0.019706132
STRONGLY	0.019371526
SIZE	0.019254929
DECLINE	0.01918024
SURFACE	0.018979657
ANNUAL	0.018895132
MODERATE	0.018652634
INDICATE	0.017980533
CONTRAST	0.017472792
DYE	0.017433229
EVIDENCE	0.017399122
MODEL	0.017273938
NATIVE	0.017272495
TEMPERATE	0.017146026
REGION	0.017090511
THERMODYNAMICS	0.017049334
CONDITIONS	0.01681726
EXPERIMENTAL	0.016326452
ASSOCIATION	0.016322793
LEAD	0.016246772
RED	0.016158137
TAXA	0.016119345
STRUCTURE	0.016048989
GEOGRAPHICAL	0.015881566
THERMODYNAMIC	0.015875464
MATRIX	0.015863216
SOCIAL	0.015247176
ISLANDS	0.015173221
MG	0.015071859
AIM	0.014977237
IDENTIFICATION	0.014927474
WATERS	0.014794559
IMPACT	0.014789568
ISLAND	0.014741583
POSITIVE	0.014710558

WHETHER	0.014537043
RECORDED	0.014344018
AVAILABILITY	0.014233608
ANIMAL	0.013785203
CHINA	0.013383848
CRITERIA	0.013365536
DELTA	0.013300814
SYSTEM	0.013259725
ANALYSES	0.013194027
PARAMETERS	0.013162776
CAPACITY	0.012917944
LINEAR	0.012848602
CANOPY	0.012783173
REVEAL	0.012689663
PHYLOGENETIC	0.012612746
COMMUNITIES	0.012512107
PHYSICS	0.012188662
ACCOUNTING	0.012058038
AVAILABLE	0.012026264
CLASSES	0.011998888
POPULATIONS	0.01183654
DIVERSITY	0.011788138
SOUTH	0.01167523
THREATENED	0.011540261
REMOVAL	0.011493755
COEFFICIENT	0.011323982
TERMS	0.011265376
STRATEGIES	0.01120245
TERRESTRIAL	0.011121956
ADDITION	0.011077302
GENUS	0.011066121
DISTRIBUTED	0.010733463
DISPERSAL	0.010703347
LOCATIONS	0.010696123
SUGGESTING	0.010592159
OCCURRENCES	0.010546822
PROTECTED	0.010497871
CORRELATED	0.010496539
GEOGRAPHIC	0.010446665
ALGORITHM	0.010405887
FINDINGS	0.010135913
VARIABILITY	0.010120617
WILD	0.009994824
PROPERTIES	0.009869727
PREDICTIONS	0.009789148
MONTH	0.009763168

DEPTH	0.009681622
PREDICTORS	0.009605697
EFFECT	0.009602478
BROAD	0.009424582
RELATIVE	0.009401733
CREATED	0.009393243
COMPONENT	0.00934124
INFORMATION	0.009272689
METE	0.009228318
ATLANTIC	0.009120762
MEDITERRANEAN	0.009077331
AFFECTING	0.009071651
ECOSYSTEMS	0.009039369
NEED	0.009029479
INDICATING	0.008837136
BEHAVIOURAL	0.008749349
SURVEY	0.008679841
FUNCTIONAL	0.008652263
CURVE	0.008583029
FEATURES	0.008521707
LIFE	0.008424353
QUARTER	0.008352266
BIRDS	0.008305711
RATIO	0.008273268
EMPLOYED	0.008012259
MODELLED	0.007998227
CENTRAL	0.007948987
CAUSED	0.007846402
BEHAVIOR	0.007800205
RESTRICTED	0.007780884
ADULT	0.007710003
COUNTRIES	0.007637723
EDGE	0.007576916
REPORTED	0.007523787
DETERMINE	0.007514769
LANDUSE	0.007441719
BIRD	0.007397289
WARMING	0.007331691
LONGTERM	0.007294494
HYPOTHESIS	0.007284488
SPACE	0.007235108
AUC	0.007210124
EQUILIBRIUM	0.007196903
MEASURE	0.007113428
DETERMINED	0.007093452
SOUTHWESTERN	0.007058124

ECOLOGICAL	0.006917409
MECHANISMS	0.006906432
SPECTRAL	0.006743862
MEAN	0.006689821
ASSUMPTIONS	0.00668802
WILDLIFE	0.006653452
PEST	0.006575546
METABOLIC	0.006488246
PUBLISHED	0.006477244
VARIABLE	0.006446734
ESTABLISHED	0.006438175
PREDICTS	0.006320218
LARGER	0.006317025
NATURAL	0.006265791
POPULATION	0.00624847
LANDSCAPES	0.006193433
COLDEST	0.006157063
GROUPS	0.006156894
NICHES	0.006153298

Table S2. Cluster 1 vs Cluster 2 Random Forest classification table. Horizontal is predicted and vertical is actual.

	**1**	**2**	**class.error**
1	603	140	0.188425
2	171	424	0.287395

Table S3. Model improvement ratio retained variables for predicting Cluster 1, Cluster 2 and Cluster 3 in Level 2.

	importance
ENTROPY	1
ECOLOGY	0.599095
HABITAT	0.252464
RANGE	0.157791
SPECIES	0.151681
DISTRIBUTION	0.149812
CLIMATE	0.109829
SUITABILITY	0.104774
SUITABLE	0.090071
CONSERVATION	0.086012
FOREST	0.077966
VARIABLES	0.064997
CURRENT	0.063146
MAXIMUM	0.06126

PREDICTED	0.060889
PRESENCE	0.058157
FUTURE	0.056302
THEORY	0.054746
ENVIRONMENTAL	0.049076
MODELS	0.044774
OCCURRENCE	0.043126
DATA	0.042447
FACTORS	0.036425
RISK	0.034973
THERMODYNAMICS	0.032813
CLIMATIC	0.031951
CHANGE	0.031663
TREES	0.030125
FOUR	0.029772
TEMPERATURE	0.028752
REGIONS	0.027304
SIGNIFICANT	0.026751
PHYSICS	0.026714
FORESTS	0.024536
COMMUNITIES	0.024297
MODELLING	0.023912
MANAGEMENT	0.023549
HABITATS	0.022946
SYSTEMS	0.021699
METHODS	0.021614
FUNCTIONAL	0.021552
STRUCTURE	0.021435
MAINLY	0.020454
SIGNIFICANTLY	0.020069
METE	0.018875
SPATIAL	0.018667
EFFECTS	0.017381
KM	0.01733
VEGETATION	0.017288
REVEALED	0.017264
NORTH	0.01684
SITES	0.01676
AFFECTED	0.016584
SOIL	0.016369
POPULATIONS	0.016016
CONDITIONS	0.015961
VARIED	0.015869
ALGORITHM	0.015816
MAXENT	0.01574
TROPICAL	0.015584

POPULATION	0.015525
INFORMATION	0.015231
METHOD	0.01515
DIVERSITY	0.014995
PLANT	0.014811
REGION	0.014456
ENTROPIES	0.014364
LANDSCAPE	0.01377
H	0.013724
COMPOSITION	0.013662
COMMUNITY	0.013614
COVER	0.013569
RESPONSES	0.013515
CHANGES	0.013319
MEASURE	0.012884
RICHNESS	0.012695
WATER	0.012506
UNITS	0.012469
FINDINGS	0.012426
ADSORPTION	0.01227
DISTINCT	0.011958
RANGES	0.011913
DENSITY	0.011616
SCENARIOS	0.011434
INDICES	0.011301
SETS	0.011187
CONDUCTED	0.010988
THERMODYNAMIC	0.010933
PROCESS	0.010805
PROCESSES	0.010634
CORRELATED	0.010582
SYSTEM	0.010533
PROJECTED	0.010528
RECORDS	0.010332
LAND	0.01031
ENERGY	0.010208
VARIATION	0.01017
NATIONAL	0.009913
LEAD	0.00981
RESEARCH	0.009593
LOCAL	0.009564
PREDICT	0.009552
ABUNDANCE	0.009432
FEATURES	0.00943
ORDER	0.009101
NICHE	0.009082

BOLTZMANN	0.008911
ECOLOGICAL	0.00878
SCALE	0.008749
INDIVIDUALS	0.008597
PRECIPITATION	0.008543
MAJOR	0.0085
STABLE	0.008481
ANALYSIS	0.008336
ASSESS	0.008308
FIELD	0.008198
SURFACE	0.008192
COASTAL	0.008122
AGRICULTURAL	0.008084
TEMPERATURES	0.008048
PRINCIPAL	0.008021
MODEL	0.007726
AVAILABILITY	0.007574
CHINA	0.007331
PARAMETERS	0.00726
DETERMINE	0.007253
TREE	0.007182
LOCATION	0.007146
DEGREES	0.007138
PERSPECTIVE	0.007121
COLLECTED	0.007061
GROUPS	0.00695
DETERMINED	0.006949
PREDICTING	0.006908
LIFE	0.006763
IMPACT	0.00667
ADDITION	0.006659
INVASIVE	0.006548
AIM	0.006491
BIODIVERSITY	0.006488
DEVELOPMENT	0.006415
CONTENT	0.006394
TAXONOMIC	0.006345
SHIFT	0.006339
MEASURED	0.006274
AVAILABLE	0.006253
PRINCIPLE	0.006107
DISTRIBUTIONS	0.006086
STRATEGIES	0.006082
MONITORING	0.006056
SELECTED	0.006028
FRAMEWORK	0.006021

BEHAVIOR	0.005828
ACCURACY	0.0058
MECHANISMS	0.005765
COMPONENT	0.005741
MICROBIAL	0.005688
METABOLIC	0.00557
OBSERVED	0.005511
GENETIC	0.00542
QUADRATIC	0.00534
PRODUCTION	0.005302
POSITIVE	0.005265
ANALYSES	0.005239
EQUILIBRIUM	0.00513
SIZE	0.005127
SAMPLING	0.004962
AFRICA	0.004918
SURVIVAL	0.004893
PREDICTIONS	0.004873
SUGGESTING	0.004836
PRESENTED	0.004791
AMERICA	0.004771
FREE	0.00477
STANDARD	0.004662
BIOMASS	0.004649
INDICATING	0.004576
TEMPORAL	0.004552
WHETHER	0.004534
ACOUSTIC	0.004533
REVIEW	0.004482
LEAF	0.004394
RATIO	0.004387
EMPLOYED	0.004374
CONSTRUCTED	0.004363
POINTS	0.004342
TOOLS	0.004333
EFFECT	0.004317
DISCUSS	0.004249
ESTIMATED	0.004232
STATISTICAL	0.004218
DRY	0.004211
MEAN	0.004206
INDICATE	0.00415
TEMPERATE	0.004099
PERFORMANCE	0.00404
FUNCTIONS	0.004008
R	0.004002

SOURCES	0.003964
CASE	0.003959
PROPERTIES	0.003945
COMPREHENSIVE	0.003943
TRADITIONAL	0.003937
ANIMAL	0.00392
INDEX	0.003919
CHARACTERISTICS	0.003912
PREDICTOR	0.003891
COMPLEXITY	0.00376
BV	0.003734
CORRELATION	0.003715
EMPIRICAL	0.003702
MOUNTAIN	0.003689
INDUSTRIAL	0.003684
INFERENCE	0.003683
SET	0.003661
TROPHIC	0.00365
GROWTH	0.003645
SCALES	0.003607
AVERAGE	0.003531
COEFFICIENTS	0.003524
VARIATIONS	0.003514
EXERGY	0.003493
IDENTIFICATION	0.00349
THROUGHOUT	0.003489
BAYESIAN	0.003477
ANALYZED	0.003462
RATHER	0.003442
GLOBAL	0.003431
DECREASED	0.003417
INTEGRATED	0.003409
SPECIFICALLY	0.003372
TERRESTRIAL	0.003351
ANALYZE	0.003302
CONTEXT	0.003262
RIVER	0.003256
CONCEPTS	0.003249
LARGEST	0.00324
THREATENED	0.003238
SEA	0.003231
PRESENT	0.003215
CONCENTRATION	0.003193
ECOSYSTEMS	0.003188
NETWORKS	0.003162
MACROECOLOGICAL	0.003135

WIDELY	0.003135

Table S4. Cluster 1, Cluster 2 and Cluster 3 Random Forest classification table. Horizontal is predicted and vertical is actual.

	**1**	**2**	**3**	**class.error**
1	287	29	37	0.186969
2	80	109	201	0.720513
3	25	74	496	0.166387

Table S5. Model improvement ratio retained variables for predicting Cluster 1, Cluster 2, Cluster 3 and Cluster 4 in Level 3.

	importance
ENTROPY	1
ECOLOGY	0.753091
SPECIES	0.195661
RANGE	0.167724
HABITAT	0.166677
DISTRIBUTION	0.138205
SUITABILITY	0.118856
CLIMATE	0.085234
THEORY	0.078446
SUITABLE	0.074598
FOREST	0.069438
CURRENT	0.065716
MODELS	0.065599
MAXIMUM	0.062298
MODEL	0.061191
VARIABLES	0.059361
ECOLOGICAL	0.058901
ENVIRONMENTAL	0.053597
DIVERSITY	0.051731
DATA	0.048407
ENTROPIES	0.041859
SYSTEMS	0.040451
OCCURRENCE	0.036281

TEMPERATURE	0.03561
CONSERVATION	0.031635
TREES	0.031158
FUTURE	0.030799
FACTORS	0.030229
PHYSICS	0.029949
SPATIAL	0.029352
CHANGE	0.028366
STRUCTURE	0.026703
MEASURE	0.026192
FOUR	0.026075
COMMUNITY	0.025427
VARIED	0.024862
METHOD	0.024774
PREDICTED	0.024625
RISK	0.024564
METHODS	0.024123
METE	0.023633
RICHNESS	0.023017
CONDITIONS	0.022359
THERMODYNAMICS	0.022246
RESEARCH	0.021898
PRESENCE	0.021342
H	0.021262
EFFECTS	0.020115
REVEALED	0.019896
DISTINCT	0.019895
NORTH	0.019484
MANAGEMENT	0.019316
MODELLING	0.019158
FORESTS	0.018908
LANDSCAPE	0.018269
HABITATS	0.017953
MODELING	0.017843
SIGNIFICANTLY	0.017628
CHANGES	0.01686
RESPONSES	0.01686
DENSITY	0.016418
MAINLY	0.016342
THERMODYNAMIC	0.016124
COMPOSITION	0.015707
KM	0.015335
CLIMATIC	0.0152
PARAMETERS	0.014947
SOIL	0.014735
TERMS	0.014285

INDICES	0.013572
ADSORPTION	0.013463
STATISTICAL	0.01322
TREE	0.013089
SIGNIFICANT	0.013079
RANGES	0.012774
FUNCTIONAL	0.012657
VEGETATION	0.01265
LOCAL	0.012567
BOLTZMANN	0.012563
ANALYSIS	0.012385
CHARACTERISTICS	0.011849
DEGREES	0.011803
FINDINGS	0.011727
SITES	0.011381
COVER	0.011241
REGIONS	0.010961
PATTERNS	0.01073
NATURAL	0.010651
SYSTEM	0.01039
PRODUCTION	0.010141
SCENARIOS	0.009943
GROWTH	0.0099
RECORDS	0.009607
DEVELOPMENT	0.009468
DYNAMICS	0.009242
BV	0.009237
PRINCIPAL	0.009076
REGION	0.009005
UNITS	0.008981
QUANTIFYING	0.00884
COMMUNITIES	0.008626
CONDUCTED	0.008612
PROPERTIES	0.008594
QUADRATIC	0.008569
AFFECTED	0.008492
PRECIPITATION	0.008439
MAJOR	0.008376
METABOLIC	0.008329
TROPICAL	0.008283
TAXONOMIC	0.00823
REFERENCE	0.008229
EVIDENCE	0.008138
ASPECTS	0.008101
PROCESS	0.007715
MECHANICS	0.00759

MONITORING	0.00754
PROJECTED	0.007356
LAND	0.007281
AVAILABILITY	0.00727
INVESTIGATED	0.007235
CONCEPTUAL	0.007152
PARTITIONING	0.007098
AGRICULTURAL	0.007001
THEORETICAL	0.006975
FUNCTIONS	0.00695
LEAD	0.006908
MATHEMATICAL	0.006906
NATURE	0.006894
SET	0.006888
APPLICATION	0.006843
PREDICTIONS	0.006821
SITE	0.006791
CORRELATED	0.006685
SETS	0.006671
ECOSYSTEMS	0.006589
PERSPECTIVE	0.006536
DECREASED	0.00653
MG	0.006486
LOCATION	0.00644
WATER	0.00642
NICHE	0.006373
SURFACE	0.00624
VARIATION	0.00608
PERFORMANCE	0.006006
APPLICATIONS	0.005918
CONSTRUCTED	0.005909
INDEX	0.005897
POPULATIONS	0.005849
RELATIONSHIP	0.005821
ESTIMATED	0.005505
ASSESS	0.005469
PROVINCE	0.005397
STABLE	0.005335
INVASIVE	0.005326
NATIONAL	0.005308
MAPS	0.005249
SPACE	0.005204
ENERGY	0.005164
RED	0.005156
SHIFT	0.00513
INFORMATION	0.005108

TOOLS	0.005101
MECHANISMS	0.005073
MEASUREMENT	0.005066
LIFE	0.005034
DELTA	0.005011
MEASURES	0.005001
SEA	0.004992
COMPLEX	0.004876
SHANNONS	0.004865
AFRICA	0.004832
CLASSES	0.004828
SELECTED	0.004776
CHINA	0.004746
FIELD	0.004687
TEMPERATE	0.004629
CONTENT	0.004628
CURRENTLY	0.004618
WIDELY	0.004615
STANDARD	0.004592
REVIEW	0.004584
ALGORITHM	0.004573
COMPONENT	0.00456
ROLE	0.004535
ANNUAL	0.004516
EXPERIMENTS	0.004491
LIVING	0.004439
CONCEPTS	0.004429
SENSITIVE	0.004407
FORM	0.004359
BEHAVIOR	0.004359
EXERGY	0.00432
KNOWLEDGE	0.004304
POPULATION	0.004276
DYE	0.004245
EMPLOYED	0.004237
ABLE	0.00423
INTERACTION	0.00423
LITERATURE	0.004222
ECONOMIC	0.004198
POSITIVE	0.004182
STATES	0.004177
ESTIMATE	0.004156
DECLINE	0.004149
ACOUSTIC	0.004145
DRY	0.00413
RATIO	0.004127

INTERACTIONS	0.004121
BEHAVIOURAL	0.004119
PREDICT	0.004115
OBSERVED	0.004097
DEFINE	0.003972
PLANT	0.003959
GRADIENTS	0.003953
EDGE	0.003936
OBJECTIVE	0.003921
AVAILABLE	0.003891
INTENSITY	0.003804
POLICY	0.003799
DEPENDENCE	0.003783
PHYSICAL	0.003773
ASSEMBLY	0.003753
ENVIRONMENT	0.00375
MAXENT	0.003679
CONCEPT	0.003676
ILLUSTRATE	0.003648
PROCESSES	0.003627
EFFICIENT	0.003623
AIM	0.003595
INDICATORS	0.003583
LIGHT	0.003581
SPECTRAL	0.003573
STRATEGIES	0.003534
VARIETY	0.00353
ORGANIC	0.003494
GROUPS	0.003478
TRADITIONAL	0.003459
TRAITS	0.003437
EFFECT	0.003436
EVOLUTION	0.003412
SUSTAINABLE	0.003409
REMOVAL	0.003398
ASSESSING	0.003388
GLOBAL	0.003386
DISCUSSED	0.00338

Table S6. Level 3 Random Forest classification table. Horizontal is predicted and vertical is actual.

	1	2	3	4	class.error

1	47	40	5	0	0.48913
2	14	148	52	47	0.43295
3	1	58	123	208	0.684615
4	1	13	73	508	0.146219

Table S7. Model improvement ratio retained variables for predicting Cluster 1, Cluster 2, Cluster 3, Cluster 4, Cluster 5, Cluster 6 and Cluster 7 in Level 4.

	importance
ENTROPY	1
ECOLOGY	0.876719
SPECIES	0.383231
HABITAT	0.166709
DISTRIBUTION	0.165574
RANGE	0.130657
SUITABILITY	0.119437
MAXIMUM	0.097692
DIVERSITY	0.091986
SUITABLE	0.091736
CLIMATE	0.090673
MODEL	0.087619
ECOLOGICAL	0.077402
MAXENT	0.075588
MODELS	0.074728
CURRENT	0.074197
AFFECTED	0.072499
FOREST	0.069196
ENVIRONMENTAL	0.067433
ENTROPIES	0.060452
VARIABLES	0.059782
DATA	0.056615
CONSERVATION	0.05567
METHODS	0.051003
SPATIAL	0.050741
MODELLING	0.041972
CHANGE	0.040108
REGIONS	0.036488
LOCATION	0.035882
SOIL	0.032499
COMMUNITY	0.029462
STRUCTURE	0.029198
FUNCTIONAL	0.029083
CLIMATIC	0.02814
TAXONOMIC	0.027976
NORTH	0.027531

PREDICTED	0.027506
FACTORS	0.02664
EFFECTS	0.025942
METHOD	0.025532
TEMPERATURE	0.025138
H	0.025071
VEGETATION	0.024896
FUTURE	0.024696
COMMUNITIES	0.024131
POPULATION	0.023722
INDICES	0.022611
ANALYSIS	0.02224
THEORY	0.021962
DEGREES	0.021568
TROPICAL	0.021123
PREDICT	0.020883
CHANGES	0.020677
MEASURE	0.02027
PROCESS	0.019928
RISK	0.019107
INVASIVE	0.019047
SET	0.018962
LOCATIONS	0.018459
SYSTEMS	0.018448
PHYLOGENETIC	0.018045
SCENARIOS	0.018044
BIODIVERSITY	0.017333
MANAGEMENT	0.017035
FUNCTION	0.016961
WATER	0.016753
TERMS	0.01663
FUNCTIONS	0.016243
BV	0.016203
ABLE	0.015996
ALGORITHM	0.015615
THERMODYNAMICS	0.015574
MONITORING	0.01522
STATISTICAL	0.014507
METE	0.013719
SIGNIFICANT	0.013674
CONCEPT	0.013466
ADSORPTION	0.013267
COMPOSITION	0.01321
MEASURED	0.01315
QUADRATIC	0.013017
RESPONSE	0.013006

EDGE	0.01292
ECONOMICS	0.012666
CHINA	0.012479
LANDSCAPE	0.012449
S	0.012436
PROPERTIES	0.012321
NATIVE	0.01222
NATURAL	0.011969
INTRODUCTION	0.011903
DISTRIBUTIONS	0.011888
MEAN	0.011797
POPULATIONS	0.011741
DENSITY	0.011541
AIM	0.011524
UNITS	0.011241
SEA	0.011142
BIOLOGICAL	0.011083
PRESENTED	0.010988
DYNAMICS	0.010913
ASSESS	0.010782
SIZE	0.010695
RESEARCH	0.01066
REVEALED	0.010549
PRECIPITATION	0.010453
APPLICATIONS	0.010184
EVALUATION	0.01
LAND	0.009987
SHIFT	0.009817
PREDICTING	0.009659
SYSTEM	0.009634
INFORMATION	0.009633
SETS	0.009527
TREE	0.009364
MODELING	0.009324
LIGHT	0.009242
COVER	0.009217
DIFFERENCE	0.009191
WILD	0.009164
THERMODYNAMIC	0.009037
INDICATE	0.008903
RICHNESS	0.008732
ROLE	0.008663
GENERATED	0.008657
EG	0.008614
CONTRIBUTE	0.008587
CANOPY	0.008529

TERRESTRIAL	0.008485
IMPACT	0.008483
HETEROGENEOUS	0.008435
TREES	0.008424
INDIVIDUAL	0.008408
PRESENT	0.008336
PATTERNS	0.008084
MAJOR	0.008057
INDEPENDENT	0.008043
MODERATE	0.008039
SITE	0.008033
AMERICA	0.007992
APPROACHES	0.007967
CONDITIONS	0.007944
PUBLISHED	0.007938
PHYSICS	0.00792
REVIEW	0.007791
ABUNDANCE	0.007762
SPACE	0.007725
FRAMEWORK	0.007701
COMPLEX	0.007578
FEATURES	0.007484
RELATIONSHIPS	0.007473
TEMPERATE	0.007443
SIGNIFICANTLY	0.007113
CAPACITY	0.007012
BOLTZMANN	0.006954
LOCAL	0.006917
NETWORK	0.006745
ECOSYSTEMS	0.006735
LIVING	0.006734
ERROR	0.00673
OCCURRENCE	0.006692
CURRENTLY	0.006659
ESTABLISHMENT	0.00655
CHARACTERISTICS	0.006529
ECOSYSTEM	0.006414
WHETHER	0.006398
PRINCIPLE	0.006251
FREE	0.006213
WATERS	0.006205
RESOURCE	0.006076
CONSISTENT	0.006005
SURFACE	0.005986
VARIATION	0.005883
SCALE	0.005808

AVERAGE	0.005789
SELECTED	0.005783
COMPREHENSIVE	0.005779
INVADED	0.00576
OBSERVED	0.00576
YEARS	0.005745
DISTINCT	0.005729
HISTORY	0.005719
SPECIFIC	0.005674
REDUCTION	0.005538
LAW	0.005522
RANGES	0.005495
EXPERIMENTS	0.005492
COMPUTATIONAL	0.005457
QUANTITATIVE	0.005434
PLOTS	0.005432
HABITATS	0.005354
DETERMINE	0.005345
ASSESSMENT	0.005335
CONSTRUCTED	0.005307
SOCIETY	0.005276
EQUATION	0.005255
PLANT	0.00525
UNDERLYING	0.005245
SDM	0.005236
TRAITS	0.005233
SOFTWARE	0.005216
CONTEXT	0.005211
STRATEGIES	0.005188
SUGGESTING	0.005164
COUNTRIES	0.005136
RESPONSES	0.005073
RATHER	0.005055
COASTAL	0.00502
SELECTION	0.005006
LINEAR	0.004939
DISPERSAL	0.004928
DEFINE	0.004921
ORDER	0.004907
ASSEMBLAGES	0.004879
INDEX	0.004864
SMALLER	0.00482
NETWORKS	0.004807
ABSENCE	0.004781
RECORDS	0.004773
LEAF	0.004763

DIVERSE	0.004761
POLICY	0.004729
FOOD	0.004722
ORGANIC	0.004676
COLLECTED	0.004667
CONCEPTUAL	0.004662
CASE	0.004605
EXTENT	0.004595
CENTRAL	0.004564
COMPONENTS	0.004539
CALCULATION	0.004536
NEED	0.004531
ANNUAL	0.004529
DISCUSS	0.004523
EVIDENCE	0.004521
ESTIMATING	0.004477
ASPECTS	0.004465
AVAILABILITY	0.004389
MATHEMATICAL	0.004362
VARIABILITY	0.004289
AROUND	0.004247
SAMPLING	0.004245
GROWING	0.004224
STATUS	0.004222
PREDICTOR	0.004213

Table S8. Level four clustering Random Forest classification table. Horizontal is predicted and vertical is actual.

	1	2	3	4	5	6	7	class.error
1	71	16	2	3	0	0	0	0.228261
2	29	50	11	26	0	9	7	0.621212
3	10	33	13	33	3	22	15	0.899225
4	12	19	9	66	7	66	37	0.694444
5	10	5	5	33	13	51	57	0.925287
6	2	6	4	44	14	93	116	0.666667
7	0	5	4	19	9	55	224	0.291139
